# Time Spent Walking and Risk of Diabetes in Japanese Adults: The Japan Public Health Center-Based Prospective Diabetes Study

**DOI:** 10.2188/jea.JE20150059

**Published:** 2016-04-05

**Authors:** Yusuke Kabeya, Atsushi Goto, Masayuki Kato, Yumi Matsushita, Yoshihiko Takahashi, Akihiro Isogawa, Manami Inoue, Tetsuya Mizoue, Shoichiro Tsugane, Takashi Kadowaki, Mitsuhiko Noda

**Affiliations:** 1 Department of Diabetes Research, National Center for Global Health and Medicine, Tokyo, Japan; 1 国立国際医療研究センター 糖尿病研究部; 2 Division of General Internal Medicine, Department of Internal Medicine, Tokai University Hachioji Hospital, Tokyo, Japan; 2 東海大学医学部付属八王子病院 総合内科; 3 Department of Public Health, Tokyo Women’s Medical University, Tokyo, Japan; 3 東京女子医科大学医学部 衛生学公衆衛生学第二講座; 4 Fiore Kenshin Clinic, Tokyo, Japan; 4 フィオーレ健診クリニック; 5 Department of Clinical Research Coordination, Center for Clinical Sciences, National Center for Global Health and Medicine, Tokyo, Japan; 5 国立国際医療研究センター 臨床研究センター 臨床研究支援部; 6 Division of Diabetes and Metabolism, Iwate Medical University School of Medicine, Morioka, Japan; 6 岩手医科大学 内科 糖尿病・代謝内科分野; 7 Department of Internal Medicine, Mitsui Memorial Hospital, Tokyo, Japan; 7 三井記念病院 内科; 8 Epidemiology and Prevention Division, Research Center for Cancer Prevention and Screening, National Cancer Center, Tokyo, Japan; 8 国立がんセンター がん予防・検診センター; 9 AXA Department of Health and Human Security, Graduate School of Medicine, The University of Tokyo, Tokyo, Japan; 9 東京大学大学院医学系研究科 健康と人間の安全保障（AXA）寄付講座; 10 Department of Epidemiology and Prevention, Center for Clinical Sciences, National Center for Global Health and Medicine, Tokyo, Japan; 10 国立国際医療研究センター 疫学予防研究部; 11 Department of Diabetes and Metabolic Diseases, The University of Tokyo, Tokyo, Japan; 11 東京大学大学院医学系研究科 糖尿病・代謝内科

**Keywords:** diabetes, time spent walking, the JPHC Diabetes study, 糖尿病, 1日の歩行時間, JPHC Diabetes study

## Abstract

**Background:**

The association between time spent walking and risk of diabetes was investigated in a Japanese population-based cohort.

**Methods:**

Data from the Japan Public Health Center-based Prospective Diabetes cohort were analyzed. The surveys of diabetes were performed at baseline and at the 5-year follow-up. Time spent walking per day was assessed using a self-reported questionnaire (<30 minutes, 30 minutes to <1 hour, 1 to <2 hours, or ≥2 hours). A cross-sectional analysis was performed among 26 488 adults in the baseline survey. Logistic regression was used to examine the association between time spent walking and the presence of unrecognized diabetes. We then performed a longitudinal analysis that was restricted to 11 101 non-diabetic adults who participated in both the baseline and 5-year surveys. The association between time spent walking and the incidence of diabetes during the 5 years was examined.

**Results:**

In the cross-sectional analysis, 1058 participants had unrecognized diabetes. Those with time spent walking of <30 minutes per day had increased odds of having diabetes in relation to those with time spent walking of ≥2 hours (adjusted odds ratio [OR] 1.23; 95% CI, 1.02–1.48). In the longitudinal analysis, 612 participants developed diabetes during the 5 years of follow-up. However, a significant association between time spent walking and the incidence of diabetes was not observed.

**Conclusions:**

Increased risk of diabetes was implied in those with time spent walking of <30 minutes per day, although the longitudinal analysis failed to show a significant result.

## Abstract (ja)

**【背景】:**

日本人成人における1日の歩行時間と糖尿病のリスクの関連を厚生労働省研究班による多目的コホート研究における糖尿病研究（JPHC Diabetes study）において調査した。

**【方法】:**

この研究では、初期調査と5年後調査が施行された。1日の歩行時間はコホート質問票によって、30分未満、30分から1時間未満、1時間から2時間未満、2時間以上に分類された。横断解析では、初期調査の参加者から既知糖尿病者を除外した26,488人を対象に、1日の歩行時間と今まで認知されていなかった糖尿病を有することの関連をロジスティック回帰分析にて解析した。次に、縦断解析では、初期調査にて糖尿病を有しておらず、5年後調査にも参加した11,101人を対象とした。初期調査における1日の歩行時間と5年後調査における糖尿病の発症との関連を同様にロジスティック回帰分析にて解析した。

**【結果】:**

横断解析では、1,058人に認知されていない糖尿病があることが判明した。歩行時間が1日2時間以上の群と比較して、30分未満の群では、認知されていない糖尿病をもつオッズが増加していた（調整後オッズ比＝1.23、95%信頼区間＝1.02-1.48）。縦断解析では、5年後調査にて612人に糖尿病の発症が確認された。しかし、1日の歩行時間と5年後調査における糖尿病の発症との間に有意な関連は認めなかった。

**【結論】:**

縦断解析では有意な結果は得られなかったが、1日の歩行時間が30分未満の者では糖尿病のリスクが上昇していることが示唆された。

## INTRODUCTION

Physical activity (PA) is an important modifiable risk factor for diabetes.^1^ A number of observational studies^2^^–^^7^ have reported that sedentary lifestyles are associated with increased risk of diabetes. The protective effect of increases in levels of PA on the incidence of diabetes has been supported by randomized controlled studies.^8^^–^^10^ Even if the intensity of exercise is moderate, the preventive effect of greater fitness on risk of diabetes has been demonstrated.^11^^,^^12^

Walking is a low- to moderate-intensity PA that is simple and practical for most people. A meta-analysis^13^ of four cohort studies^6^^,^^7^^,^^14^^,^^15^ in Western countries reported that the relative risk (RR) of developing diabetes was 0.83 (95% confidence interval [CI], 0.75–0.91) for people with regular walking compared to those with almost no walking. Evidence has been scarce in Asian populations, although one Japanese study^16^ reported that longer time spent walking is associated with decreased incidence of type 2 diabetes in middle-aged men.

In the present study, we investigated the association between time spent walking and risk of diabetes in the Japan Public Health Center-based Prospective (JPHC) Diabetes cohort, which consists of registered inhabitants in 10 public health center (PHC) areas across Japan.

## MATERIALS AND METHODS

### Study population and study design

Data from the JPHC Study, which was a large longitudinal cohort study in Japan investigating cancer, cardiovascular disease, and other lifestyle-related diseases, were used in the present study. The details of the JPHC Study have been described elsewhere.^17^ In brief, the JPHC Study was initiated in 1990 for Cohort I, and Cohort II was added in 1993. The study population consists of all registered Japanese inhabitants in 11 PHC areas who were aged 40–59 years in Cohort I and 40–69 years in Cohort II at the start of each survey. The diabetes study (the JPHC Diabetes study) was performed in 10 of the PHC areas. The initial survey was performed in 1998–1999 for Cohort II and in 2000 for Cohort I. Among the registered inhabitants of the PHC areas, those who received annual health checkups were recruited. A self-reported questionnaire specific to diabetes research and measurement of hemoglobin A1c (HbA1c) were added to their routine health checkup examinations. The 5-year follow-up survey was performed in the same way in 2003–2004 for Cohort II and in 2005 for Cohort I. The participants provided written informed consent to participate in this study. This study was approved by an ethics committee of the International Medical Center of Japan, which was a former name of the National Center for Global Health and Medicine.

We first performed a cross-sectional analysis using the data of the baseline survey to examine the association of time spent walking with the presence of unrecognized diabetes. We focused only on the association with unrecognized diabetes in the cross-sectional analysis because those with recognized diabetes might have altered their levels of physical activity due to their disease conditions. Then, we longitudinally investigated the association of time spent walking with the incidence of diabetes between the baseline and 5-year surveys.

A total of 28 363 adults participated in the baseline survey. Of the 28 363 adults who completed the baseline survey, 12 215 participated in the 5-year follow-up survey.

#### Subjects included in the cross-sectional analysis

Of the 28 363 adults at baseline, 393 were excluded because of missing data on anthropometric or laboratory measurements, and 315 were excluded because of missing data on time spent walking. In addition, 1167 participants who reported that they either presently or previously had diabetes in the questionnaire (self-reported diabetes) were excluded. Ultimately, a total of 26 488 adults were included in the cross-sectional analysis (Figure).

**Figure.**
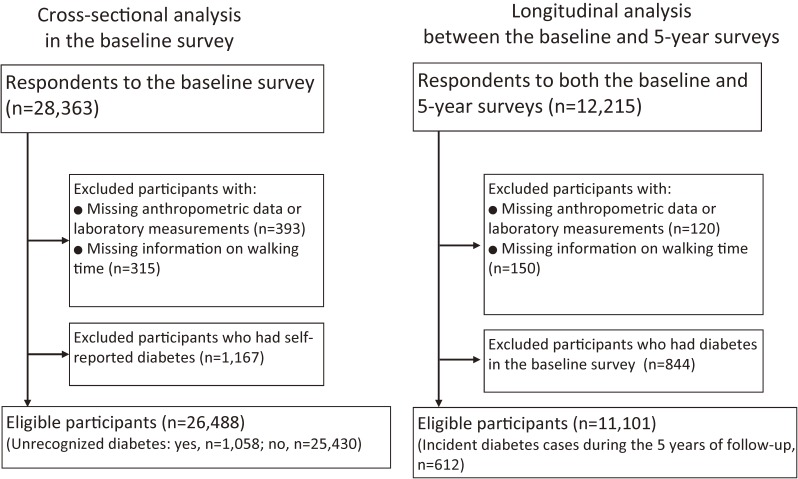
Flow chart of the study participants.

#### Subjects included in the longitudinal analysis

The longitudinal analysis was restricted to those who participated in both the baseline and 5-year surveys. Of the 12 215 participants who completed both surveys, 120 were excluded because of missing data on anthropometric or laboratory measurements, and 150 were excluded because of missing data on time spent walking. Furthermore, 844 who had diabetes at the time of the baseline survey were excluded. Ultimately, a total of 11 101 adults without diabetes were included in the longitudinal analysis (Figure).

### Measurement of time spent walking

Information on time spent walking per day was collected from the questionnaire administered at baseline. The question about time spent walking was as follows: ‘Regarding your PA level during the last 1 month, how long do you walk each day?’ The question had four possible answers: ‘less than 30 minutes’, ‘30 minutes to less than 1 hour’, ‘1 hour to less than 2 hours’ or ‘2 hours or more’. Participants were categorized into four groups according to their answers.

Although the present study did not perform a validation study, the validation of a similar questionnaire has been already reported in a Japanese population-based cohort study.^18^ In the validation study, the questionnaire asked ‘How long do you walk a day on average?’ The question had three answers: ‘less than 30 minutes’, ‘30 minutes to 1 hour’ or ‘1 hour or more’. The study reported the numbers of walking steps measured by pedometers showed linear associations with the questionnaire measurements.

### Case ascertainment of diabetes

In the cross-sectional analysis, unrecognized diabetes was diagnosed if a participant did not report having diabetes in the questionnaire (absence of self-reported diabetes) and met any of the following criteria at baseline: a fasting plasma glucose (FPG) value of 126 mg/dL or more, a casual plasma glucose value of 200 mg/dL or more, or an HbA1c value of 6.5% or more. In the longitudinal analysis, incident diabetes cases were diagnosed when a participant met any of the following criteria at the 5-year follow-up: they reported having diabetes in the questionnaire (presence of self-reported diabetes), or they had an FPG value of 126 mg/dL or more, a casual plasma glucose value of 200 mg/dL or more, or an HbA1c value of 6.5% or more. A fasting blood sample, which was defined as a sample collected ≥8 hours after the last caloric intake, was collected from 11 118 adults in the cross-sectional analysis and 3931 adults in the baseline of the longitudinal analysis. Otherwise, a blood sample was collected as a casual blood sample.

### Standardization of plasma glucose and HbA1c levels

Efforts to standardize plasma glucose levels measured at the laboratories in the PHC areas were made by the standardization committee of the JPHC study. The standardization method has been reported elsewhere.^19^ Briefly, the laboratories participated in the External Quality Control Survey by the Japan Medical Association (JMA). In the survey, four samples were sent to each laboratory. The laboratories measured samples and reported the results to the JMA. The results were evaluated in terms of the accuracy of the measurement, and the evaluation was disclosed to each laboratory. The survey revealed that the accuracy of the measurement was satisfactory.^19^ As for the HbA1c measurement, details regarding the procedure used for standardization have been also described previously.^20^ Briefly, standard samples were provided to each PHC at the time of the initial survey and the 5-year follow-up survey. The calibration procedure was conducted using the standard samples. The original standard samples were examined and approved by the Japan Diabetes Society (JDS). The procedure for HbA1c calibration used by the JDS has been described elsewhere.^21^ The averages for these standard samples were used to compute a linear regression equation using the least squares method, and the actual values were calibrated according to the regression equation. The HbA1c data were converted to equivalent values of the National Glycohemoglobin Standardization Program according to a statement made by the JDS.^22^

### Other characteristics of the study participants

Body mass index (BMI) and family history of diabetes at baseline were obtained from the questionnaire. BMI was estimated as weight (kilogram) divided by height (m) squared, the values of which were reported in the questionnaire. The validity of self-reported BMI in the JPHC Study has been examined and published previously.^23^ The self-reported BMIs were slightly lower than the measured BMIs, and the Spearman correlation coefficients were 0.89 in men and 0.91 in women. Blood pressure was measured in the right arm by trained nurses using mercury sphygmomanometers.^24^ The measurement was performed in a sitting position after at least 5 minutes rest. Family history of diabetes was defined as the presence of at least one first-degree relative with diabetes.

### Statistical analysis

We performed two analyses to investigate the relationship between daily time spent walking and risk of diabetes: 1) a cross-sectional analysis to examine the association of time spent walking with the presence of unrecognized diabetes in the baseline survey, and 2) a longitudinal analysis to examine the association of time spent walking with the incidence of diabetes between the baseline and 5-year surveys. Participants were categorized according to daily time spent walking reported in the questionnaire. For the comparison of variables among the four groups, a nonparametric test for trend across ordered groups was performed.

In the cross-sectional analysis, logistic regression was performed to calculate the odds ratios (ORs) of having unrecognized diabetes across different categories of time spent walking. The analysis was adjusted for PHC area, age, sex, BMI, family history of diabetes, and systolic blood pressure (BP). ORs were also calculated in subgroups stratified by age, sex, BMI, and family history of diabetes. To evaluate possible interactions between time spent walking and these variables, we compared models with and without the interaction term and calculated *P* values using likelihood ratio tests.

In the longitudinal analysis, the analysis was restricted to those who participated in both the baseline and 5-year surveys. The age-adjusted cumulative incidences of diabetes between the baseline survey and the 5-year survey were calculated according to the time spent walking. The age-standardization was conducted using the direct method. Incidences were standardized to a Japanese model population in 1985. Then, logistic regression analysis was performed to calculate ORs of developing diabetes across different categories of time spent walking, which was adjusted for PHC area, age, sex, BMI, HbA1c levels, family history of diabetes, and systolic BP.

Furthermore, in both the cross-sectional and longitudinal analyses, additional analyses were performed that were restricted to participants who were evaluated under fasting conditions, since the diagnosis of diabetes using casual blood samples could affect the accuracy of the case ascertainment.

Goodness of fit of the models was examined using the Hosmer-Lemeshow goodness of fit test. All analyses were performed using Stata version 11 for Windows (Stata Corp., College Station, TX, USA).

## RESULTS

### Cross-sectional analysis

Table 1 shows baseline characteristics of study participants by time spent walking per day. Details of the participants by PHC area are described in eTable 1. In the baseline survey, 1058 participants (4.0%) had unrecognized diabetes. Of the 26 488 adults, 10 807 (40.8%) reported their time spent walking was 2 hours or more a day, while 4005 (15.1%) reported less than 30 minutes. Across the walking time categories, significant trends were observed in sex ratio, the proportion of participants with family history of diabetes, BMI, systolic BP, diastolic BP, FPG levels, and HbA1c levels. The trend test in the proportion of participants with unrecognized diabetes had borderline significance. The proportion of participants with unrecognized diabetes was 4.7% in those with time spent walking of <30 minutes and 3.8% in those with time spent walking of 2 hours or more.

**Table 1.** Characteristics of study participants according to time spent walking per day (cross-sectional analysis)

	Total	Time spent walking per day				*P* for trend

		<30 min	30 min–<1 hr	1 hr–<2 hrs	≥2 hrs	
Number of subjects	26 488	4005	6197	5479	10 807	
Age, years	62.0 (7.0)	61.9 (7.3)	62.0 (7.1)	62.1 (7.1)	61.9 (6.8)	0.401
Sex, male (%)	9492 (35.8)	1460 (36.5)	2149 (34.7)	1822 (33.3)	4061 (37.6)	0.015
Family history of diabetes, yes (%)	3194 (12.1)	543 (13.6)	812 (13.1)	695 (12.7)	1144 (10.6)	<0.001
BMI, kg/m^2^	23.7 (3.2)	23.9 (3.3)	23.7 (3.2)	23.7 (3.1)	23.5 (3.1)	<0.001
Systolic BP, mm Hg	130.8 (17.8)	130.7 (17.9)	131.9 (18.1)	131.2 (17.8)	130.0 (17.5)	<0.001
Diastolic BP, mm Hg	77.6 (10.5)	78.3 (10.7)	78.3 (10.6)	77.8 (10.5)	77.0 (10.3)	<0.001
PG, mg/dL	103.8 (23.7)	104.0 (24.4)	103.5 (23.6)	103.9 (23.0)	103.8 (23.8)	0.885
PG (fasting)^a^, mg/dL	97.3 (14.0)	98.7 (17.3)	97.1 (13.4)	97.4 (12.6)	96.8 (13.5)	<0.001
HbA1c, %	5.51 (0.54)	5.49 (0.60)	5.49 (0.52)	5.52 (0.51)	5.53 (0.53)	<0.001
Unrecognized diabetes, yes (%)	1058 (4.0)	190 (4.7)	238 (3.8)	216 (3.9)	414 (3.8)	0.054

BMI, body mass index; BP, blood pressure; HbA1c, hemoglobin A1c; PG, plasma glucose.

Values are the mean (standard deviation) unless otherwise noted.

^a^A total of 11 118 participants were evaluated under fasting conditions.

Table 2 shows the number of participants with unrecognized diabetes by time spent walking and the corresponding ORs. After adjustment for PHC area, age, sex, and family history of diabetes (model 1), the OR of having diabetes for those with time spent walking of <30 minutes was 1.30 (95% CI, 1.08–1.56) compared to those with time spent walking of 2 hours or more. The observed association was not influenced by further adjustment for systolic BP. In contrast, the association became attenuated slightly after additional adjustment for BMI, although the association remained statistically significant. After additional adjustment for both systolic BP and BMI, those with time spent walking of <30 minutes still had significantly increased odds of having diabetes compared to those with time spent walking of 2 hours or more (OR 1.23; 95% CI, 1.02–1.48). The odds of having unrecognized diabetes appeared to increase as time spent walking decreased. However, the trend test became non-significant after adjustment for multiple variables, including BMI. When the analysis was restricted to the participants who were evaluated under fasting conditions in the baseline survey, the effect size of the association between time spent walking and unrecognized diabetes did not change substantially (eTable 2). Table 3 shows subgroup analyses in terms of age, sex, BMI, and family history of diabetes that were performed to detect a difference in the association. However, no significant interaction was observed among the subgroups.

**Table 2.** Time spent walking per day and unrecognized diabetes (cross-sectional analysis)

		Time spent walking per day				*P* for trend

		<30 min	30 min–<1 hr	1 hr–<2 hrs	≥2 hrs	
Unrecognized diabetes	Yes	190	238	216	414	
	No	3815	5959	5263	10 393	
Odds ratio (95% confidence interval)						
Crude odds ratio		1.25 (1.05–1.49)	1.00 (0.85–1.18)	1.03 (0.87–1.22)	1.00	0.054
Model 1		1.30 (1.08–1.56)	1.04 (0.87–1.23)	1.08 (0.91–1.28)	1.00	0.024
Model 2a		1.29 (1.07–1.56)	1.02 (0.86–1.20)	1.06 (0.89–1.26)	1.00	0.033
Model 2b		1.22 (1.01–1.47)	1.00 (0.84–1.18)	1.05 (0.88–1.25)	1.00	0.113
Model 3		1.23 (1.02–1.48)	0.99 (0.83–1.17)	1.04 (0.87–1.23)	1.00	0.113

BMI, body mass index; BP, blood pressure.

Model 1: Adjusted for public health center area, age, sex and family history of diabetes.

Model 2a: Model 1 + systolic BP.

Model 2b: Model 1 + BMI.

Model 3: Model 1 + BMI + systolic BP.

**Table 3.** Subgroup analyses of the association between time spent walking per day and unrecognized diabetes in terms of age, sex, BMI, and family history of diabetes (cross-sectional analysis)

Variable	Number of subjects	Time spent walking per day, odds ratio^a^ (95% CI)				*P* for trend	*P* for interaction

		<30 min	30 min–<1 hr	1 hr–<2 hrs	≥2 hrs		
Male	9492	1.15 (0.88–1.50)	1.07 (0.85–1.36)	1.07 (0.84–1.36)	1.00	0.301	
Female	16 996	1.27 (0.97–1.66)	0.89 (0.69–1.14)	1.00 (0.78–1.29)	1.00	0.327	0.626

Age <65 years	15 349	1.20 (0.93–1.55)	0.97 (0.76–1.22)	0.99 (0.78–1.26)	1.00	0.308	
Age ≥65 years	11 139	1.26 (0.95–1.67)	1.01 (0.78–1.30)	1.08 (0.84–1.38)	1.00	0.221	0.901

BMI <25	18 244	1.32 (1.03–1.70)	0.99 (0.79–1.25)	1.26 (1.01–1.56)	1.00	0.130	
BMI ≥25	8244	1.16 (0.87–1.54)	0.99 (0.76–1.28)	0.79 (0.60–1.05)	1.00	0.323	0.052

Family history of diabetes, Yes	3194	1.60 (1.10–2.33)	1.01 (0.70–1.45)	1.06 (0.73–1.54)	1.00	0.046	
Family history of diabetes, No	23 294	1.12 (0.90–1.40)	0.99 (0.81–1.20)	1.03 (0.85–1.26)	1.00	0.466	0.399

BMI, body mass index; CI, confidence interval.

^a^Odds ratios were adjusted for public health center area, age, sex, family history of diabetes, systolic blood pressure, and BMI except the stratifying variables.

### Longitudinal analysis

The longitudinal analysis was restricted to 11 101 participants who did not have diabetes at baseline and participated in both the baseline and 5-year surveys. Details of the cohort by PHC area are reported in eTable 3. Cohort characteristics in the baseline survey are shown in Table 4. Of the 11 101 participants, 4902 (44.2%) reported time spent walking of 2 hours or more a day, while 1444 (13.0%) reported less than 30 minutes of walking a day. As with the characteristics of participants in the cross-sectional analysis, significant trends were observed in sex ratio, the proportion of participants with family history of diabetes, BMI, systolic BP, diastolic BP, and HbA1c levels.

**Table 4.** Baseline characteristics of study participants according to time spent walking per day (longitudinal analysis)

	Total	Time spent walking per day				*P* for trend

		<30 min	30 min–<1 hr	1 hr–<2 hrs	≥2 hrs	
Number of subjects	11 101	1444	2373	2382	4902	
Age, years	62.1 (6.8)	61.9 (7.0)	62.3 (6.7)	62.4 (6.8)	62.0 (6.7)	0.509
Sex, male (%)	3691 (33.3)	449 (31.1)	744 (31.4)	735 (30.9)	1763 (36.0)	<0.001
Family history of diabetes, yes (%)	1263 (11.4)	184 (12.7)	300 (12.6)	290 (12.2)	489 (10.0)	0.001
BMI, kg/m^2^	23.7 (3.1)	23.9 (3.3)	23.7 (3.1)	23.8 (3.0)	23.5 (3.0)	0.001
Systolic BP, mm Hg	130.3 (17.4)	130.2 (17.3)	131.3 (17.6)	131.1 (17.6)	129.5 (17.1)	0.002
Diastolic BP, mm Hg	77.6 (10.3)	78.2 (10.9)	78.4 (10.6)	77.9 (10.3)	76.9 (9.9)	<0.001
PG, mg/dL	102.1 (18.5)	102.8 (18.7)	102.4 (19.2)	102.2 (18.4)	101.8 (18.2)	0.066
PG (fasting)^a^, mg/dL	94.6 (9.4)	95.1 (9.5)	94.3 (9.5)	94.7 (9.3)	94.6 (9.4)	0.723
HbA1c, %	5.44 (0.40)	5.39 (0.41)	5.42 (0.41)	5.44 (0.40)	5.45 (0.39)	<0.001

BMI, body mass index; BP, blood pressure; HbA1c, hemoglobin A1c; PG, plasma glucose.

Values are the mean (standard deviation) unless otherwise noted.

^a^A total of 3931 participants were evaluated under fasting conditions.

Table 5 shows the number of incident diabetes cases during the 5 years of follow-up according to time spent walking and the corresponding ORs. During the 5 years of follow-up, 612 participants developed diabetes, which accounted for 5.5% of the participants. The age-standardized cumulative incidence of diabetes during the 5 years of follow-up was 7.0% in those with time spent walking of <30 minutes, while it was 4.6% in those with time spent walking of 2 hours or more. Although the odds of developing diabetes appeared to increase as time spent walking decreased, the association was modest and non-significant. After adjustment for PHC area, age, sex, HbA1c levels, and family history of diabetes, the association became attenuated. The observed association became further attenuated after additional adjustment for BMI and systolic BP. When the analysis was restricted to the participants who were evaluated under fasting conditions in the 5-year survey, the modest and non-significant association did not change substantially (eTable 4).

**Table 5.** Time spent walking per day and the incidence of diabetes (longitudinal analysis)

	Time spent walking per day				*P* for trend

	<30 min	30 min–<1 hr	1 hr–<2 hrs	≥2 hrs	
Number of subjects	1444	2373	2382	4902	
Incident cases of diabetes	87	139	141	245	

Incidence of diabetes during the 5 years of follow-up^a^, %	7.0	5.8	6.4	4.6	

Odds ratios (95% confidence interval)					
Crude odds ratio	1.22 (0.95–1.57)	1.18 (0.95–1.47)	1.20 (0.97–1.48)	1.00	0.065
Model 1	1.20 (0.90–1.61)	1.17 (0.91–1.49)	1.14 (0.89–1.45)	1.00	0.149
Model 2a	1.19 (0.89–1.60)	1.14 (0.89–1.47)	1.12 (0.88–1.43)	1.00	0.177
Model 2b	1.10 (0.82–1.48)	1.13 (0.88–1.45)	1.11 (0.87–1.42)	1.00	0.374
Model 3	1.10 (0.82–1.48)	1.12 (0.88–1.44)	1.10 (0.87–1.41)	1.00	0.384

BMI, body mass index; BP, blood pressure; HbA1c, hemoglobin A1c.

^a^Standardized to Japanese model population 1985.

Model 1: Adjusted for public health center area, age, sex, HbA1c levels and family history of diabetes.

Model 2a: Model 1 + systolic BP.

Model 2b: Model 1 + BMI.

Model 3: Model 1 + BMI + systolic BP.

## DISCUSSION

The present study investigated the association between time spent walking and risk of diabetes in the JPHC Diabetes cohort. The cross-sectional analysis demonstrated that time spent walking of <30 minutes per day was significantly associated with increased odds of having unrecognized diabetes in relation to time spent walking of 2 hours or more per day. However, the longitudinal analysis did not show a protective effect of long walking time on the incidence of diabetes.

Evidence has been accumulating on the association between walking and risk of diabetes.^6^^,^^7^^,^^14^^–^^16^^,^^25^^–^^29^ Most cross-sectional studies^25^^–^^29^ have reported a non-significant inverse association between walking and odds of having diabetes. One study^28^ examining 3759 men in France, which compared the proportion of people who walk for 1 hour or more across different glycemic statuses, reported that the proportion decreased as the severity of hyperglycemia progressed, although the results of this study did not achieve statistical significance (normoglycemia, 63.7%; impaired fasting glucose, 60.7%, diabetic, 59.0%). Another study^29^ examining 5586 adults in Australia reported that the ORs for the association between walking or moderate PA for 2.5 hours or more per week and the presence of abnormal glucose metabolism was 0.96 (95% CI, 0.66–1.40) in men and 0.73 (95% CI, 0.53–1.01) in women. In contrast to these non-significant results, the present study, which examined 26 488 adults in Japan, reported a significant association between time spent walking and the presence of diabetes. Although the difference was modest, the larger sample size with sufficient diabetes cases made it possible to detect a significant association.

Some cohort studies have reported protective effects of walking on risk of diabetes. Data from the Nurses’ Health Study,^6^ which examined 70 102 female nurses in the US, showed that higher levels of metabolic equivalent (MET) score for walking were associated with a significant risk reduction in the incidence of diabetes (RR = 0.74, 95% CI = 0.59–0.93 [highest vs lowest quintile]). The similar inverse association was also reported in the study examining 37 918 male health professionals.^7^ An increase in energy expenditures of 10 METs-hours per week for walking was associated with 0.89-fold RR of the incidence of diabetes (95% CI = 0.82–0.96). As for Asian evidence, one study, which investigated 11 073 middle-aged Japanese men, reported that men who walked to work for more than 20 minutes were less likely to develop diabetes than those who did so for less than 10 minutes, with an OR of 0.73 (95% CI, 0.58–0.92).^16^ Contrary to these positive results, the longitudinal analysis in the present study, which included 11 101 Japanese adults, failed to report a significant association. In Table 5, the ORs of developing diabetes appeared higher in those with short walking time per day. However, the association became attenuated after adjustment for PHC area, age, sex, HbA1c levels, BMI, systolic BP, and family history of diabetes, suggesting that the apparent association could be confounded by these factors.

The disparity in the results between the cross-sectional analysis and the longitudinal analysis might be explained by several reasons. The possible association of time spent walking and incidence of diabetes could be too modest to be detected with the current sample size and the short follow-up period. Of the 28 363 participants in the baseline survey, only 12 215 participated in the 5-year follow-up (Figure), resulting in a large reduction in the sample size of the longitudinal analysis compared to the cross-sectional analysis. In addition, individuals included in the longitudinal analysis seemed to be healthier than those who were included in the cross-sectional analysis. Table 6 compares the baseline characteristics between those who participated in both surveys and those who participated only in the baseline survey. Those who participated in both surveys had lower levels of HbA1c, lower prevalence of diabetes, and were more active in terms of time spent walking than those who participated only in the baseline survey. Therefore, the selection of healthier individuals in the longitudinal analysis might have led to the reduction in the magnitude of the association between time spent walking and risk of diabetes. Furthermore, the baseline characteristics of participants in the longitudinal analysis (Table 4) revealed that HbA1c levels slightly increased with time spent walking per day, suggesting that participants with elevated HbA1c levels might have increased their PA levels to improve their health conditions. Such behavior could also lead to the null result in the longitudinal analysis. Meanwhile, the significant association in the cross-sectional analysis may have been superficially observed because of reverse causation. Although we excluded those with recognized diabetes from the cross-sectional analysis to minimize the reverse causation, it is still possible that individuals with diabetes spent less time walking since they were more likely to have health problems, such as heart diseases, foot problems, or impaired visual acuity. Studies with larger sample sizes or more accurate walking time measures may be required to confirm this association.

**Table 6.** Baseline characteristics of study participants who participated in the 5-year survey and those who did not

	Participants in the baseline survey			*P* for difference^a^

	Total	Participants in the baseline survey only	Participants in the two surveys	
Number of subjects	28 363	16 148	12 215	
Number of subjects after excluding participants with missing data	27 655	15 710	11 945	
Age, years	62.0 (7.0)	61.9 (7.2)	62.2 (6.8)	<0.001
Sex, male (%)	10 077 (36.4)	5983 (38.1)	4094 (34.3)	<0.001
Family history of diabetes, yes (%)	3602 (13.0)	2085 (13.3)	1517 (12.7)	0.162
BMI, kg/m^2^	23.7 (3.2)	23.7 (3.2)	23.7 (3.1)	0.055
SBP, mm Hg	131.0 (17.8)	131.2 (18.1)	130.7 (17.4)	0.018
DBP, mm Hg	77.6 (10.5)	77.6 (10.6)	77.7 (10.3)	0.771
PG, mg/dL	106.3 (29.2)	106.4 (30.1)	106.2 (28.1)	0.530
HbA1c, %	5.59 (0.70)	5.62 (0.74)	5.54 (0.65)	<0.001
Diabetes, yes (%)	2225 (8.1)	1381 (8.8)	844 (7.1)	<0.001
Unrecognized diabetes, yes (%)	1058 (3.8)	642 (4.1)	416 (3.5)	0.009
Time spent walking per day, *n* (%)				
<30 min	4202 (15.2)	2631 (16.8)	1571 (13.2)	<0.001
30 min–<1 hr	6525 (23.6)	3954 (25.2)	2571 (21.5)	
1 hr–<2 hrs	5720 (20.7)	3163 (20.1)	2557 (21.4)	
≥2 hrs	11 208 (40.5)	5962 (38.0)	5246 (43.9)	

Values are the mean (standard deviation) unless otherwise noted.

^a^Differences were tested using an unpaired *t*-test for continuous variables and a chi-square test for categorical variables.

In Japan, “National Health Promotion Movement in the 21st Century 2nd edition” was implemented in 2013 by the Ministry of Health, Labour and Welfare.^30^ One of the targets is to increase the number of daily steps taken by Japanese people. The Ministry of Health, Labour and Welfare also published the “Physical Activity Reference 2013 for health promotion,”^31^ in which moderate-intensity PA, such as walking every day for more than 60 minutes, was recommended for people aged 18 to 64 years, and low-intensity PA for 40 minutes was recommended for people aged 65 years or more. Although the present study did not find a cut-off point consistent with this public health advice, the observed increased risk of diabetes in those with time spent walking of <30 minutes may support the current public health advice.

The present study had limitations. First, as mentioned above, we did not perform a validation study on the time spent walking, which was self-reported in the present study. In the questionnaire, we asked participants about their approximate time spent walking per day over the last month. Although a similar type of a self-reported questionnaire has been found to rank the respondents satisfactorily,^18^ a questionnaire-based measurement might be limited in the ability to estimate actual time spent walking, since daily activity levels varies from day to day and the question we adopted was not specific to a certain situation (eg, leisure-time, working time, holiday, or working day). Misclassification and measurement errors could be involved in the measurement, which could dilute the association. Second, since the questionnaire used in the JPHC Diabetes study included a single-item question on time spent walking, variables related to walking intensity were not measured. In general, walking speed is higher in men than in women, and it declines with aging.^32^ Those with slower walking speed might have had a lower magnitude of association between time spent walking and risk of diabetes, since the contribution of walking to total PA might be minimal. However, the stratified analyses by age, sex, BMI, or family history of diabetes did not find a difference in the association (Table 3). Third, diabetes could be underdiagnosed in the participants who were evaluated with casual plasma glucose levels. However, additional analyses, which were restricted to those who were evaluated under fasting conditions, suggested that this issue did not substantially affect the risk estimates of the association (eTable 2 and eTable 4). Fourth, as mentioned above, selection bias could be involved in the longitudinal analysis, which weakened the generalizability of the study and could bias the results. Finally, because the data available in the present study were limited, we were not able to adjust the association for variables such as smoking status, alcohol consumption, or socioeconomic status, which could be related to both time spent walking and diabetes. The association observed in the present study could be affected by residual confounding of these factors.

The present study also has several strengths. It was based on a multi-center population-based cohort from throughout Japan. The diagnosis of diabetes was confirmed via standardized questionnaire and laboratory measurements with strict standardization.

In conclusion, we investigated the association between time spent walking and risk of diabetes in a population-based cohort in Japan. The cross-sectional analysis showed that time spent walking was significantly associated with risk of having unrecognized diabetes. On the other hand, the longitudinal analysis did not find a significant association between time spent walking and the incidence of diabetes. The results of the cross-sectional analysis might support the importance of increased walking time. However, the public health implications based on the modest association in the cross-sectional analysis and the non-significant results in the longitudinal analysis might be limited. Further research is required to confirm the protective effect of walking on the risk of diabetes.

## ONLINE ONLY MATERIALS

eTable 1. Description of the JPHC diabetes cohort in the cross-sectional analysis.

eTable 2. Cross-sectional analysis restricted to the participants who were evaluated under fasting conditions in the baseline survey.

eTable 3. Description of the JPHC diabetes cohort in the longitudinal analysis.

eTable 4. Longitudinal analysis restricted to the participants who were evaluated under fasting conditions in the 5-year survey.

Abstract in Japanese.
