# Supplementary material for: Time Spent Walking and Risk of Diabetes in Japanese Adults: The Japan Public Health Center-Based Prospective Diabetes Study
Source: J Epidemiol. 2016 Apr 5;26(4):224–32. doi: 10.2188/jea.JE20150059 (PMC4808690; doi:10.2188/jea.JE20150059)
Supplement: eTable 1. [file je-26-224-s001.pdf]

eTable 1. Description of the JPHC diabetes cohort in the cross-sectional analysis

Area	Unrecognized diabetes cases	Cohort numbers	Crude prevalence (%)	Distribution of time spent walking per day			
				< 30 min	30 min - < 1 hr	1 hr - < 2 hrs	2 hrs -
A	139	3,013	4.6	134	309	396	2,174
B	178	4,353	4.1	662	1,205	1,122	1,364
C	200	4,175	4.8	754	938	769	1,714
D	64	1,394	4.6	311	549	318	216
E	30	678	4.4	183	256	126	113
F	179	4,980	3.6	843	1,212	1,155	1,770
G	29	1,175	2.5	117	207	252	599
H	55	1,832	3.0	397	512	348	575
I	49	1,654	3.0	473	530	301	350
J	135	3,234	4.2	131	479	692	1,932
Total	1,058	26,488	4.0	4,005	6,197	5,479	10,807
