# Supplementary material for: Time Spent Walking and Risk of Diabetes in Japanese Adults: The Japan Public Health Center-Based Prospective Diabetes Study
Source: J Epidemiol. 2016 Apr 5;26(4):224–32. doi: 10.2188/jea.JE20150059 (PMC4808690; doi:10.2188/jea.JE20150059)
Supplement: eTable 2. [file je-26-224-s002.pdf]

eTable 2. Cross-sectional analysis restricted to the participants who were evaluated under fasting conditions in the baseline survey

		Time spent walking per day				*P* for
		< 30 min	30 min - <1 hr	1 hr - <2 hrs	2 hrs -	trend
Unrecognized diabetes	Yes	107	132	110	182	
	No	1,772	2,680	2,173	3,962	
Odds ratio (95% confidence interval)						
Crude odds ratio		1.31 (1.02-1.68)	1.07 (0.85-1.35)	1.10 (0.86-1.40)	1.00	0.056
Model 1		1.29 (0.99-1.67)	1.06 (0.84-1.35)	1.11 (0.87-1.43)	1.00	0.101
Model 2a		1.29 (0.99-1.67)	1.04 (0.82-1.33)	1.10 (0.86-1.41)	1.00	0.114
Model 2b		1.19 (0.91-1.54)	1.01 (0.80-1.29)	1.09 (0.85-1.40)	1.00	0.317
Model 3		1.20 (0.93-1.57)	1.01 (0.79-1.28)	1.08 (0.84-1.39)	1.00	0.284

BMI, body mass index; BP, blood pressure.

Model 1: Adjusted for public health center area, age, sex and family history of diabetes

Model 2a: Model 1 + systolic BP

Model 2b: Model 1 + BMI

Model 3: Model 1 + BMI+systolic BP
