# Supplementary material for: Time Spent Walking and Risk of Diabetes in Japanese Adults: The Japan Public Health Center-Based Prospective Diabetes Study
Source: J Epidemiol. 2016 Apr 5;26(4):224–32. doi: 10.2188/jea.JE20150059 (PMC4808690; doi:10.2188/jea.JE20150059)
Supplement: eTable 3. [file je-26-224-s003.pdf]

eTable 3. Description of the JPHC diabetes cohort in the longitudinal analysis

Area	Incident diabetes cases	Cohort numbers	Crude incidence of diabetes during the 5 years (%)	Distribution of time spent walking per day			
				< 30 min	30 min - < 1 hr	1 hr - < 2 hrs	2 hrs -
A	69	1,305	5.3	51	134	187	933
B	63	1,570	4.0	232	453	421	464
C	72	1,629	4.4	215	315	290	809
D	38	599	6.3	126	224	148	101
E	n/a	n/a	n/a	n/a	n/a	n/a	n/a
F	229	2,716	8.4	499	652	648	917
G	33	530	6.2	40	73	124	293
H	40	804	5.0	167	232	157	248
I	4	178	2.2	56	46	34	42
J	64	1,770	3.6	58	244	373	1,095
Total	612	11,101	5.5	1,444	2,373	2,382	4,902

n/a denotes "not applicable."
