# Supplementary material for: Time Spent Walking and Risk of Diabetes in Japanese Adults: The Japan Public Health Center-Based Prospective Diabetes Study
Source: J Epidemiol. 2016 Apr 5;26(4):224–32. doi: 10.2188/jea.JE20150059 (PMC4808690; doi:10.2188/jea.JE20150059)
Supplement: eTable 4. [file je-26-224-s004.pdf]

eTable 4. Longitudinal analysis restricted to the participants who were evaluated under fasting conditions in the 5-year survey

	Time spent walking per day				*P* for trend
	< 30 min	30 min - <1 hr	1 hr - <2 hrs	2 hrs -	
Number of subjects	450	818	803	1,596	
Incident cases of diabetes	34	49	43	80	
Odds ratio (95% confidence interval)					
Crude odds ratio	1.55 (1.02-2.35)	1.21 (0.84-1.74)	1.07 (0.73-1.57)	1.00	0.044
Model 1	1.48 (0.90-2.42)	1.14 (0.75-1.75)	0.97 (0.63-1.49)	1.00	0.141
Model 2a	1.46(0.89-2.40)	1.12 (0.73-1.72)	0.95 (0.62-1.47)	1.00	0.160
Model 2b	1.39 (0.84-2.28)	1.12 (0.73-1.71)	0.96 (0.62-1.49)	1.00	0.220
Model 3	1.39 (0.84-2.28)	1.11 (0.72-1.70)	0.96 (0.62-1.48)	1.00	0.225

BMI, body mass index; BP, blood pressure.

Model 1: Adjusted for public health center area, age, sex, HbA1c levels and family history of diabetes

Model 2a: Model 1 + systolic BP

Model 2b: Model 1 + BMI

Model 3: Model 1 + BMI+systolic BP
