# Supplementary material for: Time Spent Walking and Risk of Diabetes in Japanese Adults: The Japan Public Health Center-Based Prospective Diabetes Study
Source: J Epidemiol. 2016 Apr 5;26(4):224–32. doi: 10.2188/jea.JE20150059 (PMC4808690; doi:10.2188/jea.JE20150059)
Supplement: Abstract in Japanese. [file je-26-224-s005.pdf]

日本人成人における１日の歩行時間と糖尿病のリスクについて：厚生労働省研究班による多目的コホート研究における糖尿病研究（JPHC Diabetes study）の調査結果から

壁谷悠介1,2、後藤温1,3、加藤昌之4、松下由実5、高橋義彦6、五十川陽洋7、井上真奈美8,9、溝上哲也10、津金昌一郎8、門脇孝11、野田光彦1

1 国立国際医療研究センター 糖尿病研究部
2 東海大学医学部付属八王子病院 総合内科
3 東京女子医科大学医学部 衛生学公衆衛生学第二講座
4 フィオーレ健診クリニック
5 国立国際医療研究センター 臨床研究センター 臨床研究支援部
6 岩手医科大学 内科 糖尿病・代謝内科分野
7 三井記念病院 内科
8 国立がんセンター がん予防・検診センター
9 東京大学大学院医学系研究科 健康と人間の安全保障（AXA）寄付講座
10 国立国際医療研究センター 疫学予防研究部
11 東京大学大学院医学系研究科 糖尿病・代謝内科

【背景】日本人成人における 1 日の歩行時間と糖尿病のリスクの関連を厚生労働省研究班による多目的コホート研究における糖尿病研究（JPHC Diabetes study）において調査した。

【方法】この研究では、初期調査と 5 年後調査が施行された。1 日の歩行時間はコホート質問票によって、30 分未満、30 分から 1 時間未満、1 時間から 2 時間未満、2 時間以上に分類された。横断解析では、初期調査の参加者から既知糖尿病者を除外した 26,488 人を対象に、1 日の歩行時間と今まで認知されていなかった糖尿病を有することの関連をロジスティック回帰分析にて解析した。次に、縦断解析では、初期調査にて糖尿病を有しておらず、5 年後調査にも参加した 11,101 人を対象とした。初期調査における 1 日の歩行時間と 5 年後調査における糖尿病の発症との関連を同様にロジスティック回帰分析にて解析した。

【結果】横断解析では、1,058 人に認知されていない糖尿病があることが判明した。歩行時間が 1 日 2 時間以上の群と比較して、30 分未満の群では、認知されていない糖尿病をもつオッズが増加していた（調整後オッズ比＝1.23、95%信頼区間＝1.02-1.48）。縦断解析では、5 年後調査にて 612 人に糖尿病の発症が確認された。しかし、1 日の歩行時間と 5 年後調査における糖尿病の発症との間に有意な関連は認めなかった。

【結論】縦断解析では有意な結果は得られなかったが、1 日の歩行時間が 30 分未満の者では糖尿病のリスクが上昇していることが示唆された。

キーワード：糖尿病、1 日の歩行時間、JPHC Diabetes study
